# Unlocking the predictive value of post-neoadjuvant immune biomarkers in breast cancer: neutrophil-to-lymphocyte ratio (NLR) and systemic immune-inflammation index (SII)

**DOI:** 10.1007/s10549-026-07928-2

**Published:** 2026-03-16

**Authors:** María Esperanza Guirao García, Pedro Marín Rodríguez, Carmen María Servet Pérez de Lema, Noel Blaya Boluda, Pilar Sánchez Henarejos, Miguel Ángel Moya Hernández, Andrea Gottlob Pérez, Caridad Marín Hernández, Pilar de la Morena Barrio, Elisa García Garre, Elena García-Martínez, Francisco Ayala de la Peña, Antonio Piñero Madrona, Esmeralda García-Torralba

**Affiliations:** 1 Department of Medical Oncology, Hospital Universitario Morales Meseguer, 30008 Murcia, Spain; 2 Department of Medicine, Medical School, University of Murcia, 30001 Murcia, Spain; 3 Biomedical Research Institute of Murcia Pascual Parrilla–IMIB, 30120 Murcia, Spain; 4 Department of General and Digestive Surgery, Hospital Universitario Virgen de La Arrixaca, 30120 Murcia, Spain; 5 Department of Gynecology. Hospital, Universitario Virgen de La Arrixaca, 30120 Murcia, Spain; 6 Department of Medical Oncology, Hospital Universitario Virgen de La Arrixaca, 30120 Murcia, Spain; 7 Department of Surgery, Medical School, University of Murcia, 30001 Murcia, Spain

**Keywords:** Early breast cancer, Neutrophil-to-lumphocyte ratio, Systemic immune inflammation index, Complete pathological response, Prognosis

## Abstract

**Purpose:**

To evaluate the potential prognostic value of two peripheral immune biomarkers—neutrophil-to-lymphocyte ratio (NLR) and systemic immune-inflammation index (SII)—in breast cancer patients treated with neoadjuvant chemotherapy, and to assess their association with pathological complete response (pCR) and other predictive factors. In addition, to determine whether prognostic or predictive differences exist between baseline and post-neoadjuvant values of these biomarkers.

**Methods:**

We analyzed 801 women with early breast cancer treated with neoadjuvant chemotherapy, evaluating clinical and pathological data, survival outcomes, NLR (continuous and categorical) and SII.

**Results:**

Baseline NLR was significantly higher in younger patients, in those with positive pathological nodes, and in the HER2 + /HR − subtype, while baseline SII was elevated in the triple-negative subtype. Post-neoadjuvant chemotherapy (post-NCT) NLR and SII showed only weak associations with estrogen receptor expression, yet both were independently associated with pCR (post-NCT NLR: OR = 0.91; 95% CI: 0.84–0.98; *p* = 0.02; post-NCT SII: OR = 0.65; 95% CI: 0.47–0.89; *p* = 0.008). Neither biomarker showed a significant impact on overall or progression-free survival.

**Conclusion:**

Post-treatment NLR and SII may reflect chemotherapy-induced immune changes and are associated with pathological complete response, but their additional predictive value is uncertain, and no prognostic impact was observed.

**Supplementary Information:**

The online version contains supplementary material available at 10.1007/s10549-026-07928-2.

## Introduction

Despite advances in its treatment, breast cancer (BC) remains a major global health concern [1] [2]. Neoadjuvant chemotherapy (NCT) is a standard treatment for early-stage HER2-positive (HER2 +) and triple-negative breast cancer (TNBC) and is the preferred strategy for patients with locally advanced and inflammatory BC [3, 4]. NCT improves the likelihood of breast-conserving surgery, enables dynamic response monitoring, and informs adjuvant treatment decisions through risk stratification. It also serves as a prognostic tool for short- and medium-term outcomes and provides a valuable framework for investigating predictive biomarkers and accelerating drug development [5].

Risk stratification is critical for patients undergoing NCT, as residual pathological disease significantly impacts postoperative management. Pathological complete response (pCR) is a strong surrogate marker for recurrence risk, disease-free survival (DFS), and overall survival (OS), particularly in aggressive subtypes such as HER2 + BC and TNBC. However, despite achieving pCR, recurrence occurs in up to 30–40% of cases within five years. Therefore, the identification of additional prognostic factors is essential to distinguish patients at increased risk of relapse, even after pCR is achieved [6]. The immune system plays a key role in tumor immunosurveillance, so numerous immunological parameters, such as tumor-infiltrating lymphocytes (TILs), are under investigation [6, 7].

In the absence of a single optimal biomarker, easily measurable and dynamic indicators such as peripheral blood immune biomarkers have garnered growing interest. Among these, the neutrophil-to-lymphocyte ratio (NLR) is one of the most widely evaluated in BC. While previous studies associate high NLR values with increased BC related mortality and shorter DFS as an independent prognostic factor [8–19], others have failed to confirm these associations for either DFS [20, 21] or OS [12, 18] in contemporary cohorts. Similarly, correlations between NLR and tumor size or nodal status remain inconsistent [22, 23].

The predictive utility of NLR for achieving pCR following NCT has also yielded conflicting findings. Again, some studies support its role [15], while other works did not find any association [19, 20, 24]. Consequently, newer composite indices such as the SII have been proposed as more integrative markers of systemic inflammation. SII incorporates platelet counts into its formula, calculated as (platelets × neutrophils)/lymphocytes [25–27]. In BC patients treated with NCT, low baseline SII values have been associated with longer DFS and OS [28, 29], whereas high SII values have been linked to adverse prognostic factors, including increased risk of axillary nodal involvement [30].

Emerging evidence also suggests that chemotherapy-induced immune modulation may alter peripheral biomarker profiles during NCT, potentially contributing to the heterogeneity observed across studies. This has led to growing interest in post-treatment biomarker measurements as potentially more reliable prognostic and predictive indicators. Several studies have highlighted the value of tracking these indices dynamically, reporting that lower post-NCT SII values are associated with a higher likelihood of achieving pCR [31–34]. However, most studies on the SII are in Eastern populations [35–38], which may not fully represent the prognostic behavior of this index in Western populations [39, 40].

In relation to these findings, the absolute lymphocyte count (ALC) has also been proposed as a clinically meaningful peripheral immune biomarker. Beyond its independent prognostic relevance, ALC is an integral component of both the neutrophil-to-lymphocyte ratio (NLR = absolute neutrophil count [ANC]/ALC) and the systemic immune-inflammation index (SII = [ANC × platelets]/ALC). Accordingly, lower ALC values will mathematically translate into higher NLR and SII values, which have been interpreted as surrogates of systemic inflammation and a more immunosuppressed host environment. In breast cancer, baseline lymphopenia (ALC < 1000/uL) has been associated with poorer prognosis across multiple settings, and higher pre-treatment ALC has been linked to a greater likelihood of pCR, particularly in TNBC and HER2-positive disease [41–43]. From a prognostic perspective, lymphopenia has been associated with worse OS and higher recurrence risk after neoadjuvant treatment, including in patients achieving pCR and in those with residual disease [44, 45].

In this study, we evaluated a Western cohort of patients with localized BC treated with NCT. Our objectives were to assess the prognostic value of post-NCT biomarker values, determine their predictive value for pCR, and explore their associations with clinical and tumor-related variables.

## Material and methods

### Cohort assembly, diagnosis, and treatment

This study analyzed a prospective observational cohort from two academic centers, comprising 801 women diagnosed with early-stage breast cancer between 2009 and 2019, all of whom were treated with NCT followed by surgery. Of these, 454 (56.7%) patients were treated at University Hospital Morales Meseguer and 347 (43.3%) at University Hospital Virgen de la Arrixaca.

Eligibility criteria included female sex, histologically confirmed stage I–III breast cancer, and the availability of both pre-treatment (baseline) and post-NCT complete blood counts. Peripheral biomarker assessment required blood tests conducted prior to the initiation of NCT and within two weeks before surgery.

Diagnosis and treatment followed standard clinical practice during the study period [46]. Chemotherapy regimens were classified as second-generation (e.g., doxorubicin–cyclophosphamide or docetaxel–cyclophosphamide) or third-generation (sequential or concurrent administration of anthracyclines and taxanes).

Primary endpoints included the NLR, SII, disease free survival, and overall survival, as defined below. The pCR was defined as the absence of residual invasive tumor in breast and axillary nodes (ypT0/ypTis ypN0).

The study was approved by the Clinical Research and Trials Committee of University Hospital Morales Meseguer (Internal code: EST08/21) and conducted in accordance with the ethical principles of the Declaration of Helsinki.

## Neutrophil-to-lymphocyte ratio (NLR) and systemic immune-inflammation index (SII) calculation

Routine hematological parameters were retrieved from laboratory databases, using the most recent complete blood counts obtained before NCT initiation and before surgery (after completion of NCT), with a maximum interval of two weeks. NLR was calculated as the ratio between the absolute neutrophil count and the absolute lymphocyte count, both at baseline and post-NCT. The SII was computed using the same blood count samples, applying the formula: (platelet count × neutrophil count)/lymphocyte count.

## Sample size and power estimation

Assuming a 90% censoring rate and a two-sided alpha of 0.05, the study (n = 801) had 80% power to detect a hazard ratio (HR) ≥ 2.0 for overall survival between two equal-sized groups (1:1). Biomarker distributions (NLR and SII) were analyzed as both continuous and dichotomous variables, with median values used as cut-off points for dichotomization.

## Statistical analysis

Descriptive statistics for categorical variables were presented as proportions. The Shapiro–Wilk test was used to assess normality of continuous variables. Normally distributed variables were reported as means ± standard deviations (SD), while non-normally distributed variables were expressed as medians and interquartile ranges (IQR).

Proportions and ordinal variables were compared using Pearson’s χ^2^ test. Differences in means were assessed using the Student’s t-test (for parametric data) or the Mann–Whitney U test (for non-parametric data). Associations between biomarkers and ordinal variables (e.g., tumor subtype) were evaluated with the Kruskal–Wallis test. Correlations between biomarkers (NLR and SII) and continuous variables (e.g., Ki-67, estrogen/progesterone receptor expression) were examined using Spearman’s rho; rho values < 0.20 were considered non-significant. The association between pCR and NLR and SII (both as continuous variables) was assessed with multivariate logistic regression models adjusting for potential confounders.

DFS was defined as the time from the first NCT cycle to the first occurrence of invasive locoregional or distant relapse or breast cancer–related death. Distant recurrence free interval (DRFI) was defined as the time from the first NCT cycle to the first occurrence of distant recurrence or breast cancer–related death; patients experiencing locoregional recurrence without documented prior distant recurrence were censored at the date of locoregional recurrence. OS was defined as the time from treatment initiation to death from any cause. Survival analyses were performed using Kaplan–Meier curves and compared with log-rank tests. Median follow-up was estimated via the inverse Kaplan–Meier method.

The prognostic impact of clinical and biological variables was assessed using univariate and multivariate Cox proportional hazards models. Variables included in the multivariate models were selected based on theoretical plausibility and previous literature. Treatment-related variables were included to ensure model validity across treatment conditions. Biomarkers were modeled as continuous variables. To avoid multicollinearity, variables with strong correlations (|r|≥ 0.5) or statistically significant associations (p < 0.05) were excluded.

All p-values were two-sided, with statistical significance set at p < 0.05. Analyses were conducted using R version 4.2.3 and RStudio (version 2023.03.0).

## Results

### Patient characteristics and treatment

A total of 801 women with early-stage breast cancer were included in the study. Baseline patient and tumor characteristics are summarized in Table 1. The median age at diagnosis was 49 years (IQR: 42–59). The most frequent tumor subtypes were HR +/HER2 − (46.6%) and HR +/HER2 + (23.7%), followed by TNBC (16.9%) and HER2 +/HR − (12.7%).

**Table 1:** Baseline patient and tumor characteristics

	Total cohort (*n* = 801)
Hospital	
Hospital 1	454 (56.7%)
Hospital 2	347 (43.3%)
Age	
Median [Q1, Q3]	49.0 [42.0, 59.0]
Menopausal status	
Premenopausal	447 (55.8%)
Postmenopausal	351 (43.8%)
NA	3 (0.4%)
Subtype	
HR +/HER2-	373 (46.6%)
HR +/HER2 +	190 (23.7%)
HER2 +/HR-	102 (12.7%)
TNBC	135 (16.9%)
NA	1 (0.1%)
Ki67	
Median [Q1, Q3]	35.0 [21.0, 60.0]
NA	35 (4.4%)
cT	
cT1	61 (7.6%)
cT2-4	712 (88.9%)
NA	28 (3.5%)
cN	
cN0	259 (32.3%)
cN1	274 (34.2%)
cN2-3	226 (28.2%)
NA	42 (5.3%)
ypT	
ypT0	226 (28.2%)
ypT1	288 (36%)
ypT2-4	229 (28.4%)
ypTis	42 (5.2%)
NA	16 (2.2%)
ypN	
ypN0	407 (50.8%)
ypN1	156 (19.5%)
ypN2-3	122 (15.2%)
ypN1mi	41 (5.1%)
ypNx	38 (4.7%)
NA	37 (4.6%)
pCR	
No	552 (68.9%)
Yes	249 (31.1%)
CT	
2nd generation CT	154 (19.2%)
3rd generation CT	647 (80.8%)

Regarding clinical staging, most tumors were classified as cT2 (2–5 cm), accounting for 55.2% of cases. Clinical lymph node involvement was present in 62% of patients (cN1: 34.2%, cN2–3: 28.2%).

Third-generation chemotherapy regimens (sequential or concurrent anthracycline-taxane combinations) were used in the majority of patients (80.8%). Residual tumor stage after NCT was ypT0 in 28.2%, ypT1 in 36%, and ypT2–4 in 28.4%. Pathological nodal status post-NCT was ypN0 in 50.8%, with 19.5% showing ypN1 involvement and 15.2% ypN2–3. The overall pCR rate after NCT was 31.1%.

*CT* chemotherapy. 2nd generation *CT,* taxane or anthracycline-based regimens (*TC* docetaxel-cyclophosphamide, *AC* doxorubicin-cyclophosphamide), 3rd generation *CT* sequential/concurrent anthracyclines and taxanes, (*TAC* docetaxel, doxorubicin, cyclophosphamide, weekly Paclitaxel-Doxorubicin/cyclophosphamide), *ER* Estrogen receptor, *PR* Progesterone receptor, *NA* not available.

## Peripheral blood immune biomarkers (NLR, SII): distribution

Peripheral immune biomarkers were analyzed as continuous variables. The median post-NCT neutrophil-to-lymphocyte ratio was 2.37 (IQR: 1.64–3.33), while the median baseline NLR was 1.94 (IQR: 1.51–2.74). A weak but statistically significant correlation was observed between baseline and post-NCT NLR (Spearman’s ρ = 0.26; p < 0.001).

Similarly, the median post-NCT SII was 0.61 (IQR: 0.39–0.89), compared to a baseline median of 0.47 (IQR: 0.33–0.71). A comparable correlation was observed between baseline and post-treatment SII (Spearman’s ρ = 0.25; p < 0.001). As expected, pre- and post-NCT NLR values showed strong positive correlations with their corresponding SII values (Spearman’s ρ = 0.80; p < 0.001) (Supplementary Table 1).

The median post-NCT ALC was 1,275 cells/µL (IQR: 900–1,689), compared with a baseline median of 1,930 cells/µL (IQR: 1,525–2,400). The proportion of patients with lymphopenia (ALC < 1,000 cells/µL) increased from 4.6% at baseline to 34.9% post-NCT. Baseline and post-NCT ALC were weakly but significantly correlated (Spearman’s ρ = 0.35; p < 0.001). Given the way these indices are calculated, baseline and post-NCT ALC showed moderate inverse correlations with the corresponding NLR values (Spearman’s ρ = −0.56 and −0.54, respectively; p < 0.001) and SII values (Spearman’s ρ = −0.45 and −0.40, respectively; p < 0.001).

## Peripheral blood immune biomarkers (NLR, SII): association with clinical and pathological variables

**Fig. 1:**
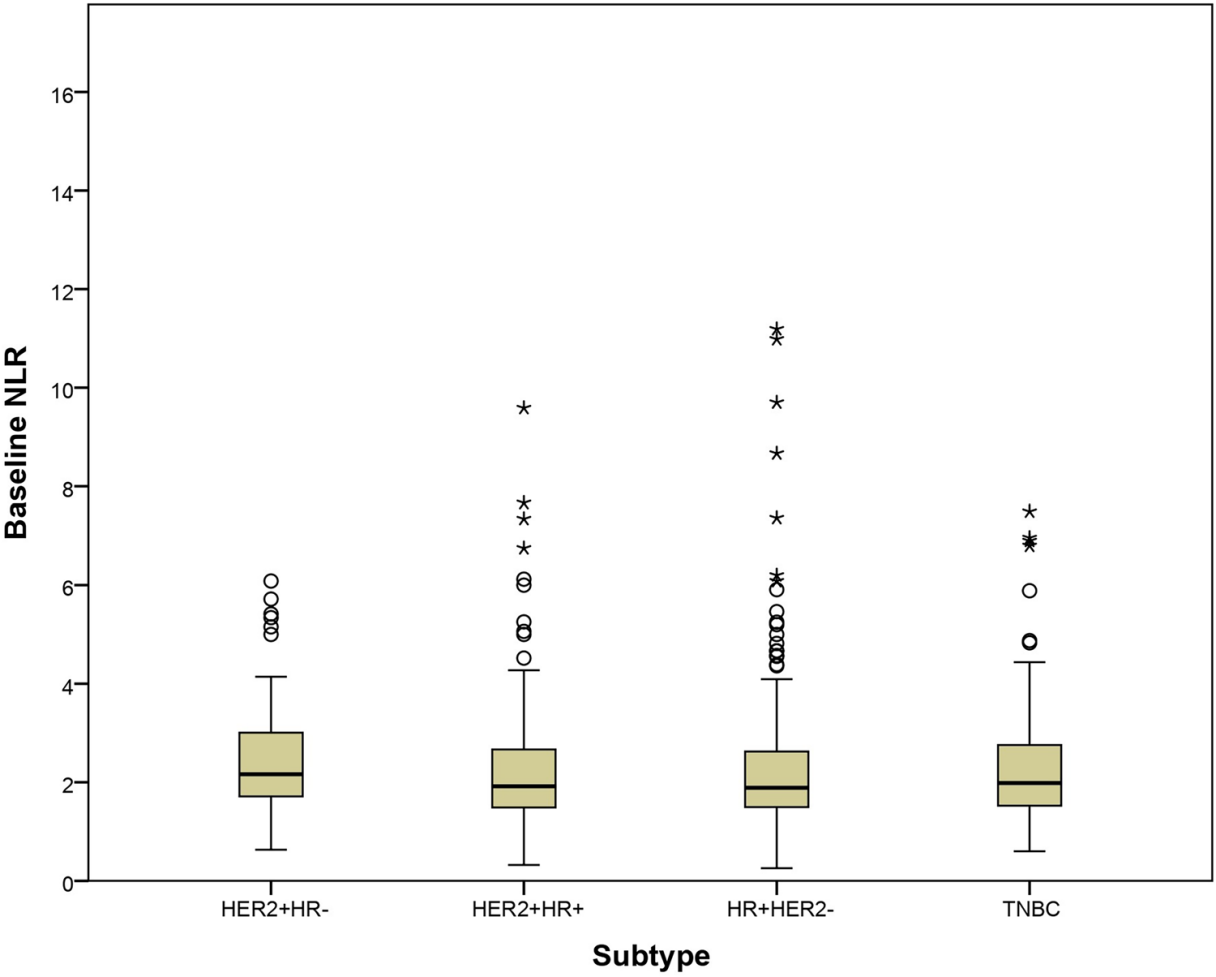
Values of baseline (pre-NCT) NLR according to tumour subtypes

Baseline NLR values were significantly higher in younger and premenopausal patients (p = 0.01 and p < 0.001, respectively), as well as in those without positive pathological nodes (p = 0.02) and in the HER2 +/HR − subtype (Kruskal–Wallis χ^2^ = 11.4; p = 0.04) (Supplementary Table 1 and 2, and Fig. 1). Regarding baseline SII, it was significantly elevated in patients with triple-negative breast cancer (Kruskal–Wallis χ^2^ = 76; p = 0.02), and a weak negative correlation were observed with estrogen receptor (ρ = –0.12; p < 0.001) and progesterone receptor (ρ = –0.10; p = 0.004) expression (Supplementary Table 1 and 2).

**Fig. 2:**
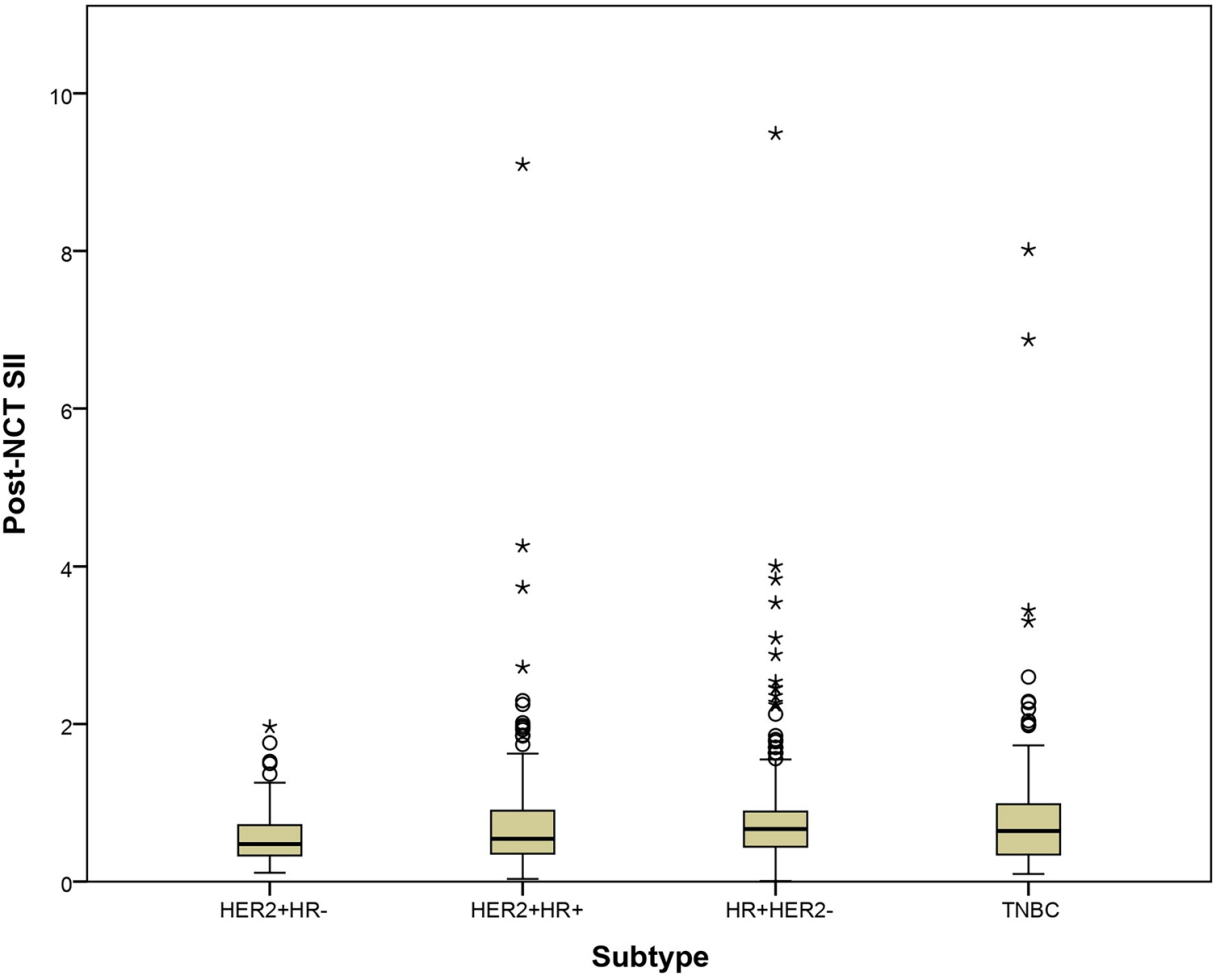
Values of post-NCT SII according to tumour subtypes

Post-NCT NLR demonstrated a weak positive correlation with estrogen receptor (ER) expression (Spearman’s ρ = 0.12; p = 0.001) and was significantly higher in the HR +/HER2 − subtype (Kruskal–Wallis χ^2^ = 14.8; p = 0.01) (Supplementary Table 3 and 4, and Figure S1). Regarding post-NCT SII was also weakly associated with ER positivity (Spearman’s ρ = 0.09; p = 0.007) and showed higher levels in the HR +/HER2 − subtype (Kruskal–Wallis χ^2^ = 17.5; p = 0.002) (Supplementary Table 3 and 4, and Fig. 2). No other significant associations with clinical or pathological variables were observed.

## Association of NLR and SII with pathological complete response

A multivariate logistic regression model was used to assess the predictive value of post-NCT NLR and SII for pCR, as continuous variables, adjusting for potential confounders. In the adjusted model, both biomarkers were independently associated with pCR: post-NCT NLR (OR = 0.91; 95% CI: 0.84–0.98; *p* = 0.02) and post-NCT SII (OR = 0.65; 95% CI: 0.47–0.89; *p* = 0.008) (Tables 2 and Table 3).

**Table 2:** Multivariate logistic regression model including post-NCT NLR for pathological complete response

	Beta	OR (95% CI)	*p* value
Baseline NLR	0.98	0.86–1.12	0.80
Post-NCT NLR	0.91	0.84–0.98	0.02
Ki-67	1.01	1.01–1.02	< 0.001
Lymph node involvement (cN +)	0.78	0.55–1.12	0.177
Postmenopausal status	0.87	0.61–1.23	0.43
Progesterone receptor positive (PgR +)	0.26	0.18–0.37	< 0.001
Anthracycline- and taxane-based chemotherapy	1.24	0.79–1.94	0.346

**Table 3:** Multivariate logistic regression model including post-NCT SII for pathological complete response

	Beta	OR (95% CI)	*p* value
Baseline SII	0.93	0.61—1.42	0.738
Post-NCT SII	0.65	0.47–0.89	0.008
Ki-67	1.01	1.01–1.02	< 0.001
Lymph node involvement (cN +)	0.77	0.54–1.09	0.149
Postmenopausal status	0.875	0.62–1.24	0.451
Progesterone receptor positive (PgR +)	0.26	0.18–0.37	< 0.001
Anthracycline- and taxane-based chemotherapy	1.23	0.78–1.93	0.370

In contrast, no significant associations were found between baseline NLR or SII and pCR (baseline NLR: OR = 0.98; 95% CI: 0.86–1.12; baseline SII: OR = 0.93; 95% CI: 0.61–1.42). Likewise, ALC was not significantly associated with pCR at baseline or post-NCT. Results were also non-significant when ALC was dichotomized using a cutoff of < 1,000 cells/µL (baseline lymphopenia: OR = 0.44; 95% CI, 0.13–1.50; post-NCT lymphopenia: OR = 0.81; 95% CI, 0.52–1.26).

Additional variables significantly associated with pCR included higher Ki-67 and PR negativity (Table 2 and Table 3).

## Association of NLR and SII with survival outcomes

In the subgroup of 454 patients from Hospital 1 with long-term follow-up available, the association of post-NCT biomarkers with OS, DRFI and DFS was assessed. Biomarkers were analyzed both as continuous variables (Cox regression) and dichotomized using median values (log-rank test).

**Fig. 3:**
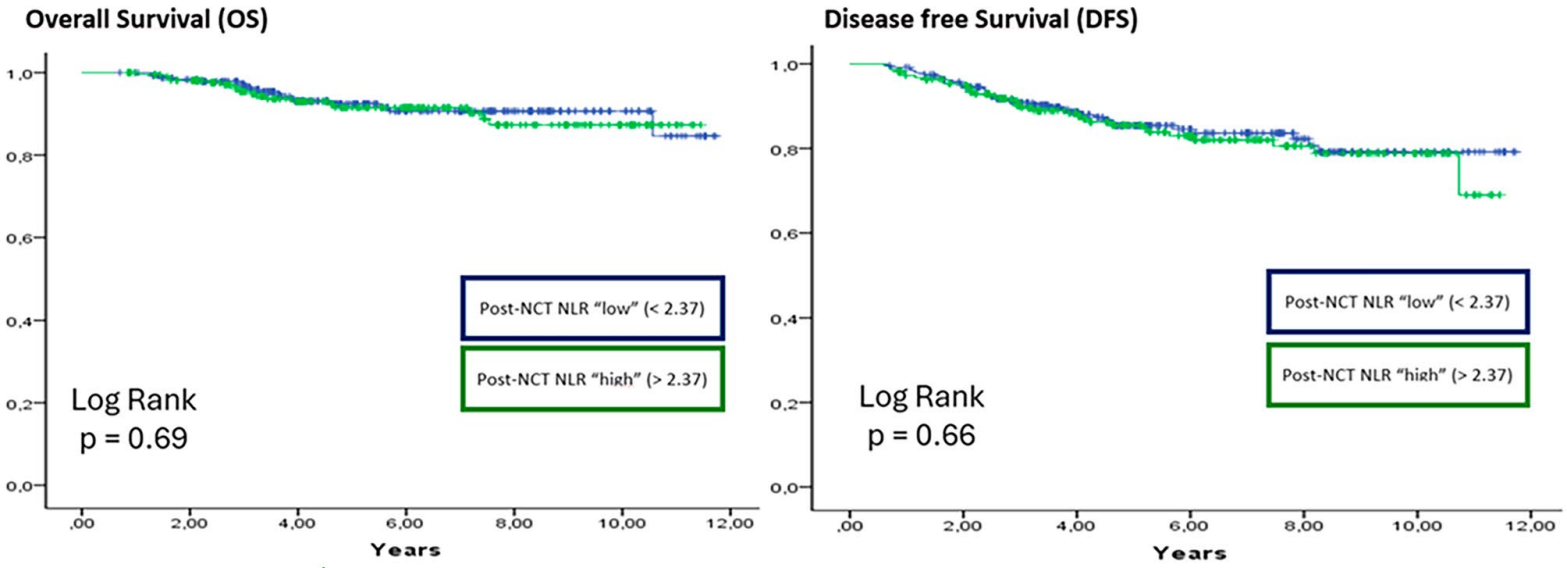
Kapplan Meier curve and log-rank test for overall survival and disease free survival in the group of patients with post-NCT NLR. *N* = 454

**Fig. 4:**
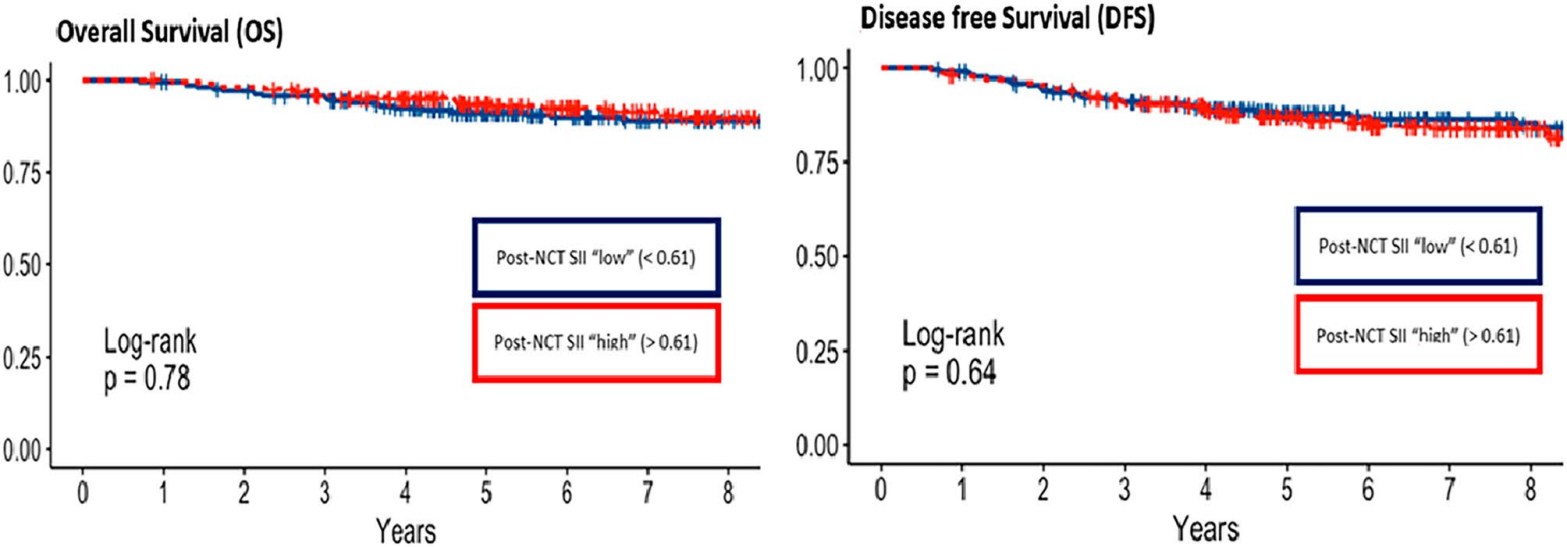
Kapplan Meier curve and log-rank test for for overall survival and diseasefree survival in the group of patients with post-NCT SII. N = 454

After a median follow-up of 7.71 years (IQR: 7.33–8.08), post-NCT NLR was not associated with OS, DRFI, or DFS in the overall cohort (continuous/dichotomized: OS *p* = 0.70/0.69; DRFI *p* = 0.63/0.73; DFS *p* = 0.67/0.66) (Fig. 3). Similarly, post-NCT SII showed no association with OS, DRFI, or DFS (continuous/dichotomized: OS *p* = 0.90/0.78; DRFI *p* = 0.80/0.96; DFS *p* = 0.70/0.64) (Fig. 4). Post-NCT ALC and lymphopenia were also not associated with OS, DRFI, or DFS (ALC: OS *p* = 0.93; DRFI *p* = 0.59; DFS *p* = 0.52; lymphopenia: OS *p* = 0.83; DRFI *p* = 0.12; DFS *p* = 0.14). Among patients achieving pCR, post-NCT SII remained non-significant for OS and DFS in both continuous and dichotomized analyses (OS *p* = 0.30/0.20; DFS *p* = 0.80/0.80).

## Discussion

The immune profile of patients with cancer differs substantially from that of healthy individuals, often characterized by a baseline immunosuppressive and pro-tumor phenotype [47]. Consequently, increasing attention is being paid to the assessment of immune biomarkers in cancer patients, including post-treatment values as potential predictors of response. In this work, we have evaluated the association between post-NCT NLR, ALC and SII values and both pCR and survival in early and locally advanced breast cancer.

Among circulating immune biomarkers, the NLR has been one of the most extensively investigated in BC. However, results remain inconsistent regarding its predictive value for pCR and its association with survival outcomes [8–24]. Attempts to refine peripheral biomarkers by incorporating additional parameters – such as the systemic immune-inflammation index, which includes platelet counts [25, 26]—have also yielded heterogeneous findings. As a result, neither NLR nor SII have been incorporated into routine clinical practice. Notably, recent studies have suggested that biomarker levels measured after NCT may provide more reliable prognostic and predictive information than baseline values [31–34]. Building on this hypothesis, our study focused on the association between post-NCT NLR and SII values and both pCR and survival outcomes, as well as their potential correlations with established clinicopathological variables. Consistent with prior reports [33, 34], we observed that lower post-NCT values of NRL and, mainly, of SII, were significantly associated with higher pCR rates (OR 0.91 and 0.65, respectively). In contrast, no such association was found for baseline values. However, previous studies investigating the potential association between these two baseline peripheral biomarkers have reported a linear correlation between them, as well as a significant association of their values with overall survival. In those studies, the SII demonstrated superior prognostic capacity compared to the NLR, as reflected by a higher area under the curve (AUC) in ROC analyses [26, 27].

Moreover, considerable heterogeneity exists in the cut-off values used across studies, with thresholds for high NLR ranging from 1.9 to 4.0 [9]; further complicating data interpretation. Several confounding factors may contribute to baseline variability in these indices—including systemic inflammation, infection, comorbidities, steroid use, and other unmeasured host-related factors [9]. Our findings support the hypothesis that post-treatment biomarker levels are likely less influenced by baseline variability, because they may be influenced by the antitumor therapy received and the subsequent biological response, reflecting treatment-induced immune modulation and its relationship with response.

Despite the relatively long median follow-up period of 7.7 years, neither post-NCT NLR, ALC nor SII were associated with DFS, DFRI or OS, even in the subgroup of patients achieving pCR. Although limited in number, previous studies have reported conflicting results regarding the association between post-treatment biomarker levels and long-term outcomes [31–34]. These inconsistencies underscore the need for standardized methodologies and extended longitudinal analyses, as well as the pursuit of more refined biomarkers, such as circulating immune cell populations, cytokine levels, or other molecules involved in immune regulation.

In terms of correlations with other prognostic features, although the two biomarkers (NLR, SII) were strongly correlated with each other, their associations with tumor characteristics and treatment response displayed distinct patterns. The results suggest that baseline NLR, in line with earlier findings [9], is associated with adverse prognostic factors (it was higher in younger, premenopausal women and in those with lymph node involvement). In this sense, it appears to reflect the baseline inflammatory status without providing additional prognostic value beyond those clinical features. In contrast, baseline SII seems to be more closely related to tumor biology (it was most elevated in patients with triple-negative breast cancer) rather than with tumor stage or burden. Other studies have reported variable associations between SII and clinicopathological parameters, including Ki-67 and nodal involvement, further underscoring the need for continued investigation [31].

Post-NCT NLR showed a weak association with ER expression, particularly within the HR +/HER2- subtype. This observation should be interpreted cautiously, as this subtype was the most prevalent in our cohort. Previous studies have similarly reported limited associations between NLR and tumor subtype [11, 13, 15]. Post-NCT SII also demonstrated a weak positive correlation with the HR +/HER2- subtype. Given that this is the most common and prognostically favorable BC subtype, the clinical relevance of this association remains uncertain.

Our study has several strengths, including a relatively large sample size and a follow-up period comparable to that of similar studies (typically ranging from 250 to 2,000 patients). Moreover, this study was conducted on a cohort of Western patients, a population that has been largely underrepresented in previously published studies, which may imply potential predictive and prognostic differences in the value of these biomarkers compared to prior research, most of which was conducted in East Asian populations [35–40]. However, some limitations must be acknowledged. First, our cohort included patients with early-stage disease, a population generally characterized by favorable outcomes, which may limit the ability to detect significant survival differences (although it is precisely where prognostic stratification is most relevant). Second, biomarker values were assessed only at baseline and after NCT completion, without intermediate time points. Recent studies have proposed that dynamic changes in immune indices during treatment—such as the difference between post- and pre-NCT values—may provide more informative prognostic insights [33], therefore early dynamic changes in these biomarkers may potentially be useful for tailoring treatment duration; however, this hypothesis requires validation in future studies. While we explored this approach in preliminary analyses, results were inconclusive and are not presented here. Additionally, several potential confounders that can influence immune parameters—such as infections, steroid exposure, or use of granulocyte colony-stimulating factors—were not uniformly recorded, despite attempts to standardize treatment protocols.

Taken together, our results could indicate the existence of an association between pCR to specific changes in peripheral immunity, which could be associated with the prognostic impact of pCR either by the eradication of disease preventing the emergence of resistance or by a surrogacy association with the sensitivity of micrometastatic disease to treatment. Future studies with larger cohorts, longer follow-up, and serial immune monitoring are warranted to further clarify the prognostic and predictive value of these biomarkers and facilitate their integration into clinical practice.

## Conclusions

In this study, we evaluated the prognostic and predictive value of two peripheral immune biomarkers—NLR and SII—in patients with early-stage breast cancer treated with neoadjuvant chemotherapy. Our findings support the association of post-treatment values of NLR and SII with pathological complete response, while no association was observed with long-term survival outcomes. These results suggest that immune biomarkers measured after treatment may better reflect tumor-immune dynamics and response to therapy than baseline values. The lack of post-NCT NLR and SII impact on prognosis warrants further research in well-characterized cohorts with longitudinal sampling to improve the understanding of post-treatment immune balance. Our results also suggest that the post-treatment setting might be particularly appropriate in the search of circulating biomarkers with potential clinical applicability in personalizing neoadjuvant treatment strategies.

## Supplementary Information

Below is the link to the electronic supplementary material.

Supplementary file1 (DOCX 309 KB)

## Data Availability

The data that support the findings of this study are available from the corresponding author upon reasonable request.
