# Supplementary material for: Unlocking the predictive value of post-neoadjuvant immune biomarkers in breast cancer: neutrophil-to-lymphocyte ratio (NLR) and systemic immune-inflammation index (SII)
Source: Breast Cancer Res Treat. 2026 Mar 16;216(3):26. doi: 10.1007/s10549-026-07928-2 (PMC12992397; doi:10.1007/s10549-026-07928-2)
Supplement: Supplementary file 1 — Supplementary file1 (DOCX 309 KB) [file 10549_2026_7928_MOESM1_ESM.docx]

**TITLE**: **Unlocking the predictive value of post-neoadjuvant immune biomarkers in breast cancer: neutrophil-to-lymphocyte ratio (NLR) and** **systemic immune-inflammation index (SII)**

**AUTHORS:** María Esperanza Guirao García^1,2,3^, Pedro Marín Rodríguez^3,4^, Carmen María Servet Pérez de Lema^3,5^, Noel Blaya Boluda^1,2,3^, Pilar Sánchez Henarejos^3,6^, Miguel Ángel Moya Hernández^1,3^, Andrea Gottlob Pérez^1,2,3^, Caridad Marín Hernández^3,4^, Pilar de la Morena Barrio^1,2,3^, Elisa García Garre^1,2,3^, Elena García-Martínez^1,3^, Francisco Ayala de la Peña^1,2,3^, Antonio Piñero Madrona^3,4,6^, Esmeralda García-Torralba^1,2,3†^

1. Department of Medical Oncology, Hospital Universitario Morales Meseguer, Murcia, 30008, Spain.
2. Department of Medicine, Medical School, University of Murcia, Murcia, 30001, Spain.
3. Biomedical Research Institute of Murcia Pascual Parrilla–IMIB, Murcia, 30120, Spain.
4. Department of General and Digestive Surgery, Hospital Universitario Virgen de la Arrixaca, Murcia, 30120, Spain.
5. Department of Gynecology. Hospital Universitario Virgen de la Arrixaca, Murcia, 30120, Spain.
6. Department of Medical Oncology, Hospital Universitario Virgen de la Arrixaca, Murcia, 30120, Spain.
7. Department of Surgery, Medical School, University of Murcia, Murcia, 30001, Spain.

† e-mail: esmeralda.garciat@um.es

*Supplementary Table 1. Association of post-NCT NLR with other biological and pathologic variables*

|  |  | **ER%** | **PR%** | **Age** | **Ki-67** | **Baseline SII** | **Pos-NCT SII** | **Baseline NLR** | **Post-NCT NLR** |
| --- | --- | --- | --- | --- | --- | --- | --- | --- | --- |
| **ER%** | Correlation coefficient * | 1.000 | 0.626 | 0.090 | -0.288 | -0.129 | 0.096 | -0.064 | 0.120 |
|  | Sig. (bilateral) | . | 0.000 | 0.011 | 0.000 | 0.000 | 0.007 | 0.074 | 0.001 |
|  | N | 789 | 788 | 789 | 759 | 789 | 774 | 789 | 774 |
| **PR%** | Correlation coefficient * | 0.626 | 1.000 | -0.050 | -0.271 | -0.102 | 0.017 | -0.041 | 0.046 |
|  | Sig. (bilateral) | 0.000 | . | 0.164 | 0.000 | 0.004 | 0.630 | 0.252 | 0.198 |
|  | N | 788 | 789 | 789 | 758 | 789 | 774 | 789 | 774 |
| **Age** | Correlation coefficient * | 0.090 | -0.050 | 1.000 | -0.115 | -0.079 | 0.011 | -0.090 | 0.016 |
|  | Sig. (bilateral) | 0.011 | 0.164 | . | 0.001 | 0.025 | 0.750 | 0.011 | 0.644 |
|  | N | 789 | 789 | 801 | 766 | 801 | 786 | 801 | 786 |
| **Ki-67** | Correlation coefficient * | -0.288 | -0.271 | -0115 | 1.000 | 0.040 | -0.030 | 0.065 | -0.050 |
|  | Sig. (bilateral) | 0.000 | 0.000 | 0.001 | . | 0.263 | 0.419 | 0.070 | 0.169 |
|  | N | 759 | 758 | 766 | 766 | 766 | 751 | 766 | 751 |
| **Baseline SII** | Correlation coefficient * | -0.129 | -0.102 | -0.079 | 0.040 | 1.000 | 0.248 | 0.830 | 0.166 |
|  | Sig. (bilateral) | 0.000 | 0.004 | 0.025 | 0.263 | . | 0.000 | 0.000 | 0.000 |
|  | N | 789 | 789 | 801 | 766 | 801 | 786 | 801 | 786 |
| **Post-NCT SII** | Correlation coefficient * | 0.096 | 0.017 | 0.011 | -0.030 | 0.248 | 1.000 | 0.240 | 0.856 |
|  | Sig. (bilateral) | 0.007 | 0.630 | 0.750 | 0.419 | 0.000 | . | 0.000 | 0.000 |
|  | N | 774 | 774 | 786 | 751 | 786 | 786 | 786 | 786 |
| **Baseline NLR** | Correlation coefficient * | -0.064 | -0.041 | -0.090 | 0.065 | 0.830 | 0.240 | 1000 | 0.263 |
|  | Sig. (bilateral) | 0.074 | 0.252 | 0.011 | 0.070 | 0.000 | 0.000 | . | 0.000 |
|  | N | 789 | 789 | 801 | 766 | 801 | 786 | 801 | 786 |
| **Post-NCT NLR** | Correlation coefficient * | 0.120 | 0.046 | 0.016 | -0.050 | 0.166 | 0.856 | 0.263 | 1.000 |
|  | Sig. (bilateral) | 0.001 | 0.198 | 0.644 | 0.169 | 0.000 | 0.000 | 0.000 | . |
|  | N | 774 | 774 | 786 | 751 | 786 | 786 | 786 | 786 |

**Spearman’s Rho.*

*Supplementary Table 2. Association of baseline NLR and SII and other biological and pathologic variables*

| **Variables** | **Baseline NLR (median, Q1-Q3)** | **p-value** | **Baseline SII (median, Q1-**  **Q3)** | **p-value*** |
| --- | --- | --- | --- | --- |
| **Menopausal status** |  | 0.000 |  | 0.001 |
| Premenopausal | 2.05 (1.59 – 2.83) |  | 0.31 (0.36 – 0.75) |  |
| Postmenopausal | 1.81 (1.41 – 2.56) |  | 0.45 (0.31 – 0.66) |  |
| **ER status** |  | 0.153 |  | 0.000 |
| Positive ER | 1.90 (1.50 – 2.66) |  | 0.46 (0.31 – 0.69) |  |
| Negative ER | 2.06 (1.58 – 2.80) |  | 0.52 (0.39 – 0.80) |  |
| **RP status** |  | 0.140 |  | 0.000 |
| Positive RP | 1.93 (1.50 – 2.63) |  | 0.44 (0.31 – 0.68) |  |
| Negative RP | 1.98 (1.55 – 2.80) |  | 0.51 (0.38 – 0.76) |  |
| **HER2 status** |  | 0.153 |  | 0.452 |
| Positive HER2 | 2.03 (1.58 – 2.85) |  | 0.47 (0.34 – 0.72) |  |
| Negative HER2 | 1.93 (1.50 – 2.70) |  | 0.47 (0.32 – 0.71) |  |
| **cN** |  | 0,204 |  | 0.031 |
| cN0 | 2.01 (1.58 – 2.80) |  | 0.49 (0.36 – 0.79) |  |
| cN1/2/3 | 1.93 (1.50 – 2.67) |  | 0.46 (0.32 – 0.69) |  |
| **ypN** |  | 0.020 |  | 0.03 |
| ypN0 | 2.03 (1.56 – 2.83) |  | 0.48 (0.35 – 0.77) |  |
| ypN1/2/3 | 1.88 (1.48 – 2.58) |  | 0.47 (0.31 – 0.66) |  |

* *Mann Whitney U-test.*

Supplementary Table 3*. Association of post-NCT NLR and SII with other biological and pathologic variables*

| **Variables** | **Post-NCT NLR (median, Q1-Q3)** | **p-value** | **Post-NCT SII (median, Q1-**  **Q3)** | **p-value** |
| --- | --- | --- | --- | --- |
| **Menopausal status** |  | 0.940 |  | 0.704 |
| Premenopausal | 2.29 (1.67 – 3.37) |  | 0.63 (0.38 – 0.91) |  |
| Postmenopausal | 2.41 (1.58 – 3.33) |  | 0.60 (0.39 – 0.87) |  |
| **ER status** |  | 0.012 |  | 0.032 |
| Positive ER | 2.46 (1.69 – 3.46) |  | 0.63 (0.41 – 0.90) |  |
| Negative ER | 2.21 (1.49 – 3.14) |  | 0.56 (0.33 – 0.87) |  |
| **RP status** |  | 0.354 |  | 0.698 |
| Positive RP | 2.40 (1.67 – 3.45) |  | 0.61 (0.39 – 0.86) |  |
| Negative RP | 2.28 (1.60 – 3.30) |  | 0.63 (0.38 – 0.92) |  |
| **HER2 status** |  | 0.001 |  | 0.000 |
| Positive HER2 | 2.08 (1.50 – 3.27) |  | 0.53 (0.35 – 0.84) |  |
| Negative HER2 | 2.50 (1.74 – 3.43) |  | 0.67 (0.42 – 0.92) |  |
| **cN** |  | 0.084 |  | 0.632 |
| cN0 | 2.22 (1.60 – 3.14) |  | 0.60 (0.37 – 0.86) |  |
| cN1/2/3 | 2.48 (1.67 – 3.45) |  | 0.62 (0.40 – 0.90) |  |
| **ypN** |  | 0.104 |  | 0.301 |
| ypN0 | 2.24 (1.58 – 3.39) |  | 0.60 (0.37 – 0.88) |  |
| ypN1/2/3 | 2.50 (1.69 – 3.35) |  | 0.63 (0.41 – 0.91) |  |

* *Mann Whitney U-test.*

Supplementary Table 4*. Association of post-NCT SII with other biological and pathologic variables*

|  |  | **Ki-67** | **Baseline NLR** | **Post-NCT NLR** | **ER%** | **PR%** | **Baseline SII** | **Post-NCT SII** |
| --- | --- | --- | --- | --- | --- | --- | --- | --- |
| **Ki-67** | Correlation coefficient | 1.000 | 0.065 | -0.050 | -0.288 | -0.271 | 0.040 | -0.030 |
|  | Sig. (bilateral) | . | 0.070 | 0.169 | 0.000 | 0.000 | 0.263 | 0.419 |
|  | N | 766 | 766 | 751 | 759 | 758 | 766 | 751 |
| **Baseline NLR** | Correlation coefficient | 0.065 | 1.000 | 0.263 | -0.064 | -0.041 | 0.830 | 0.240 |
|  | Sig. (bilateral) | 0.070 | . | 0.000 | 0.074 | 0.252 | 0.000 | 0.000 |
|  | N | 766 | 801 | 786 | 789 | 789 | 801 | 786 |
| **Post-NCT NLR** | Correlation coefficient | -0.050 | 0.263 | 1.000 | 0.120 | 0.046 | 0.166 | 0.856 |
|  | Sig. (bilateral) | 0.169 | 0.000 | . | 0.001 | 0.198 | 0.000 | 0.000 |
|  | N | 751 | 786 | 786 | 774 | 774 | 786 | 786 |
| **ER%** | Correlation coefficient | -0.288 | -0.064 | 0.120 | 1.000 | 0.626 | -0.129 | 0.096 |
|  | Sig. (bilateral) | 0.000 | 0.074 | 0.001 | . | 0.000 | 0.000 | 0.007 |
|  | N | 759 | 789 | 774 | 789 | 788 | 789 | 774 |
| **PR%** | Correlation coefficient | -0.271 | -0.041 | 0.046 | 0.626 | 1.000 | -0.102 | 0.017 |
|  | Sig. (bilateral) | 0.000 | 0.252 | 0.198 | 0.000 | . | 0.004 | 0.630 |
|  | N | 758 | 789 | 774 | 788 | 789 | 789 | 774 |
| **Baseline SII** | Correlation coefficient | 0.040 | 0.830 | 0.166 | -0.129 | -0.102 | 1.000 | 0.248 |
|  | Sig. (bilateral) | 0.263 | 0.000 | 0.000 | 0.000 | 0.004 | . | 0.000 |
|  | N | 766 | 801 | 786 | 789 | 789 | 801 | 786 |
| **Post-NCT SII** | Correlation coefficient | -0.030 | 0.240 | 0.856 | 0.096 | 0.017 | 0.248 | 1.000 |
|  | Sig. (bilateral) | 0.419 | 0.000 | 0.000 | 0.007 | 0.630 | 0.000 | . |
|  | N | 751 | 786 | 786 | 774 | 774 | 786 | 786 |

**Spearman’s Rho*


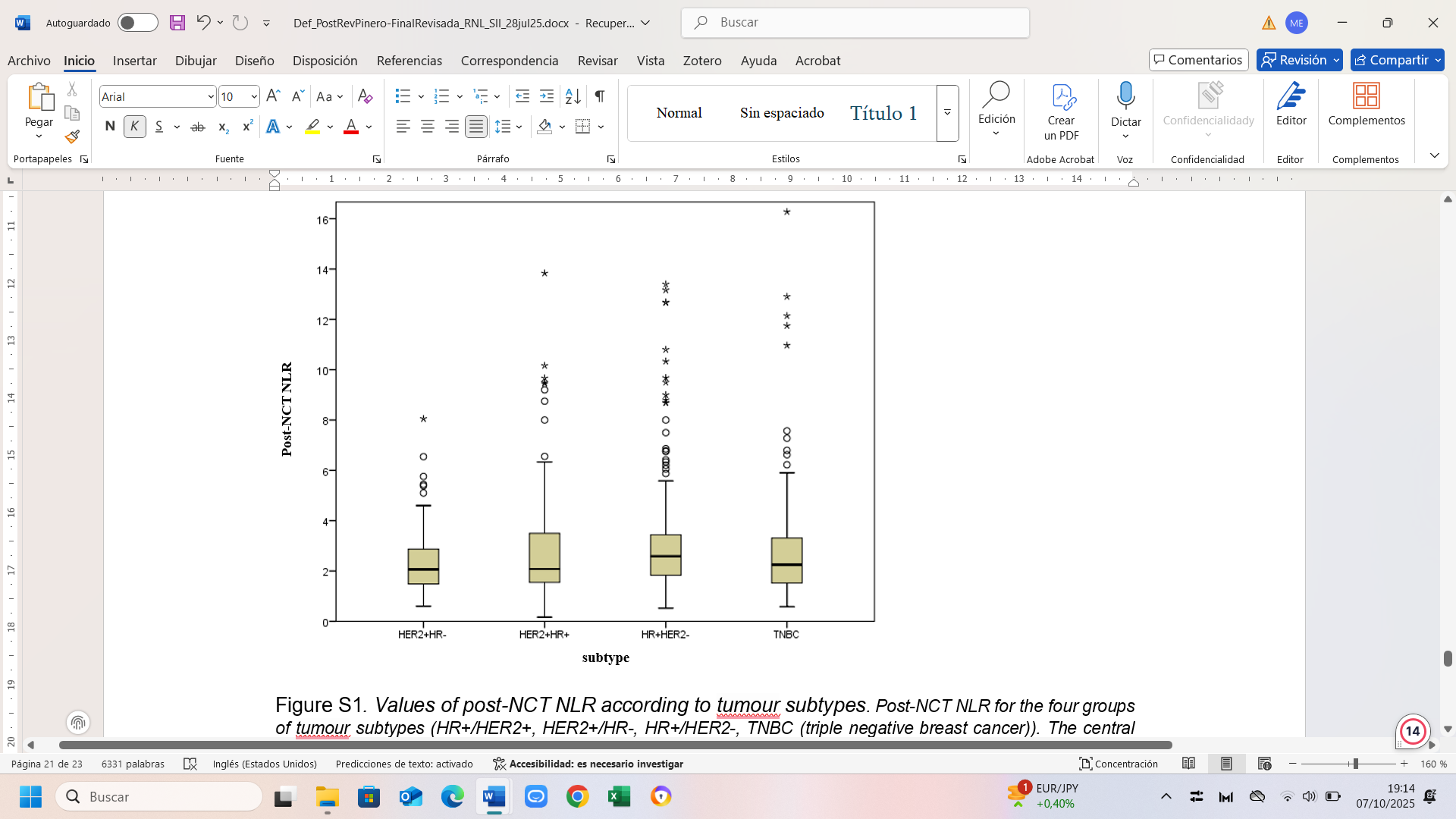


Figure S1*. Values of post-NCT NLR according to tumor subtypes.*

*Post-NCT NLR for the four groups of tumor subtypes (HER2+/HR+, HER2+/HR-, HR+/HER2-, TNBC (triple negative breast cancer)). The central line in each boxplot corresponds to the median value of baseline NLR; black dots correspond to outliers; error bars represent + 1.5 IQR. Kruskal-Wallis test.*
